# Incisional hernia recurrence after open elective repair: expertise in abdominal wall surgery matters

**DOI:** 10.1186/s12893-019-0569-6

**Published:** 2019-08-07

**Authors:** J. A. Pereira, A. Bravo-Salva, B. Montcusí, S. Pérez-Farre, L. Fresno de Prado, M. López-Cano

**Affiliations:** 1 Servicio de Cirugía General y del Aparato Digestivo, Parc de Salut Mar. Hospital del Mar. P, Marítim 23-25, 08003 Barcelona, Spain; 2 Departament de Ciències Experimentals i de la Salut, Universitat Pompeu Fabra, Dr. Aiguader 80, 08003 Barcelona, Spain; 3 Servicio de Cirugía General y Digestiva, Hospital Vall d’Hebrón, Passeig Vall d’Hebrón 119-129, 08035 Barcelona, Spain; 4 Departament de Cirurgia. Vall d’Hebrón, Universitat Autònoma de Barcelona, Passeig Vall d’Hebrón 119-129, 08035 Barcelona, Spain

**Keywords:** Incisional hernia, Open repair, Expertise, Recurrence, Abdominal wall unit

## Abstract

**Background:**

Recurrence after incisional hernia repair is one of the major problems related with this operation. Our objective is to analyze the influence of abdominal wall surgery expertise in the results of the open elective repair of incisional hernia.

**Methods:**

We have compiled the data of a cohort of patients who received surgery for an incisional hernia from July 2012 to December 2015 in a University Hospital. Data were collected prospectively and registered in the Spanish Register of Incisional Hernia (EVEREG). The short- and long-term complications between the groups of patients operated on by the Abdominal Wall Surgery (AWS) unit and groups operated on by surgeons outside of the specialized abdominal wall group (GS) were compared.

**Results:**

During the study period, a total of 237 patients were operated on by the open approach (114 AWS; 123 GS). One hundred seventy-five patients completed a median follow-up of 36.6 months [standard deviation (SD) = 6]. Groups were comparable in terms of age, sex, body mass index (BMI), comorbidities, and complexity of hernia. Complications were similar in both groups. Patients in the AWS group presented fewer recurrences (12.0% vs. 28.9%; *P* = 0.005). The cumulative incidence of recurrence was higher in the GS group [log rank 13.370; *P* < 0.001; odds ratio (OR) = 37.8; 95% confidence interval (CI) = 30.3–45.4]. In the multivariate analysis, surgery performed by the AWS unit was related to fewer recurrences (OR = 0.19; 95%CI = 0.07–0.58; *P* < 0.001).

**Conclusion:**

Incisional hernia surgery is associated with better results in terms of recurrence when it is performed in a specialized abdominal wall unit.

Incisional hernia repair is one of the most frequent procedures performed in General Surgery [1]. Although it is a common operation, real-world evidence shows high figures of incisional hernia recurrence (IHR) [2]. The Danish hernia registry reported a 12.7% IHR in 3212 patients [3]. A Swedish registry reported up to 23% IHR when the hernia width was greater than 3 cm and in onlay mesh repair [4] and, a Spanish registry reported 20.7% IHR after 1-year follow-up, especially in hernias that were previously repaired (18.1% primary vs. 30.6% recurrence) [5].

Risk factors for IHR have been related to patient characteristics (e.g., older age, obesity, diabetes, smoking, immunosuppression) [6], hernia characteristics (e.g., transverse diameter, location, recurrence, mesh) [6], and surgical performance (e.g., experience, dexterity, previous training) [7, 8].

Surgical expertise in abdominal wall surgery as a risk factor for IHR has been poorly studied [7, 8]. However, in other fields such as colorectal or bariatric surgery, a positive relationship of specialization and better results has been found [9–12]. Also, in heart surgery, expertise has been related to better outcomes [13].

In the previous context, the aim of our study is to analyze the influence of surgeon specialization and expertise in abdominal wall surgery in incisional hernia outcomes, especially in recurrence.

## Patients and methods

This is an observational cohort study of patients who received an open elective incisional hernia repair in a single center between July 2012 and December 2015. Data were compiled prospectively into the Spanish Incisional Hernia Registry (EVEREG) [14].

Patients were distributed in two groups: patients operated on by surgeons non-specialized in abdominal wall surgery (GS group), and patients who were operated on in the Abdominal Wall Surgery Unit (AWS group).

The AWS unit is comprised of a senior surgeon who specializes in abdominal wall surgery, a fellow, and a resident. In our General Surgery department 300 groin hernia, 150 primary or recurrent ventral hernia and 70 incisional hernia repairs are performed per year. The AWS unit performs approximately 150 inguinal hernia, 100 primary or recurrent ventral hernias and 50 incisional hernias elective repairs per year. All surgeries included in the AWS group were performed by the senior surgeon, or at least with the senior surgeon as first assistant. According to this parameters, our abdominal wall surgeon and center would achieve specialist required parameters published by different national organizations. [15–17] The rest of the surgeries performed were done by the GS group, conformed by 10 surgeons specialized in other fields. Due to the absence of criteria for the definition of specialization in abdominal wall surgery, we established some for this category, according to that suggested previously: high surgical volume and more than 5 years of surgical dedication to abdominal wall surgery [18].

All the risk factors related to abdominal wall complications were recorded [6]: age, sex, body mass index (BMI), surgical risk score from the American Society of Anesthesiologists (ASA), presence of chronic obstructive pulmonary disease (COPD), diabetes mellitus (DM), history of cancer, and immunosuppression.

Incisional hernias were classified according to location, width and length of hernia defect, and by complexity as defined by Slater [19] in three grades: severe, moderate, or minor.

The Carolinas Equation for Determining Associated Risks (CeDAR) score [20], which predicts complications and cost previous to surgery, was calculated for all patients.

Space mesh placement, type of mesh or fixation selection is chosen according to individual hernia cases and preference of each surgeon. AWS unit tendency during study period was to use anterior abdominal wall component separation and sub lay mesh position as primary option and composite meshes are only used when doubt of possible bowel contact is suspected. Despite hernia repair preferences each case is studied to give an individual tailored treatment. Variables related to technique (i.e., use of mesh, mesh position, type of mesh, associated procedures, and use of abdominal wall component separation) and variables related to the operation (i.e., time of surgery, intraoperative and postoperative complications, and length of stay) were also collected. Postoperative complications were stratified using the Clavien–Dindo system [21].

After hospital discharge, patients were followed up at 1 and 6 months, 1 year, and 2 years. In each visit, the presence of complications (e.g., chronic pain, chronic infection, presence of bowel fistula, and mesh removal) was assessed and recorded. Recurrence was evaluated on each follow-up visit by clinical exploration, and in case of doubt, an image exploration was indicated (ultrasound or computed tomography).

All patients and data analyzed in our study are registered and available for properly follow-up into Spanish Incisional Hernia Registry database, EVEREG [14]. We included all patients with at least one year follow-up for long-term outcomes and recurrence analysis. Time to hernia recurrence was calculated as the time from surgery to diagnosed recurrence.

Statistical analysis was performed using the SPSS v.20.0 (IBM Inc., Rochester, MN) statistical package. Quantitative variables are presented as the mean ± standard deviation (SD), and categorical variables are presented as proportions. The association between qualitative variables was assessed using contingency tables (Chi-square test and Fisher test, when necessary) and the quantitative test using the Student t test for unpaired data or the Mann-Whitney U test when necessary. The normality of the distribution of quantitative variables was checked using the Kolmogorov-Smirnov test. Statistical significance was established at *p* < 0.05. The odds ratio (OR) of hernia recurrence was calculated for each group with its confidence intervals (CI).

Multivariate analysis of risk factors for incisional hernia was performed. Predictive capacity of each variable and its independence were analyzed using survival curves for Incisional hernia incidence was estimated by a non-parametric Kaplan-Meier method. A Cox proportional hazards regression model was used to select risk of IH recurrence.

The development of the study was performed following international guidelines of clinical investigation (Ethics Code and Helsinki Declaration) and according to legal regulations for confidentiality and personal data. The study protocol was approved by the local ethics committee.

## Results

A total of 237 patients received an open elective incisional hernia repair during the study period, with 114 patients in the AWS group and 123 in the GS group. No differences were found in preoperative characteristics between the groups, except a higher rate of previous cancer surgery in the GS group (GS 28.3% vs. AWS 19%; *P* = 0.01). In addition, the probability of surgical complications graded by the CeDAR equation score [20] was higher in patients in the AWS group (GS 15.33 vs. AWS 18.96; *P* = 0.02) (Table 1).

**Table 1:** Preoperative Patient characteristics and comorbidities

	AWS *N* = 114	GS *N* = 123	*P* value
Age, years (SD)	63.8 (12.5)	66.2 (10.9)	0.45
Age > 70 N (%)	46 (40.4)	54 (43.9)	0.33
Sex M/F N (%)	69 (60.5)/45 (39.5)	62 (50.4)/61(49.6)	0.11
BMI kg/m^2^ (SD)	29.1(4.7)	29.0 (4.3)	0.55
BMI > 25 N (%)	93 (81.6)	92 (74.8)	0.15
BMI > 30 N (%)	46 (40.4)	50 (40.7)	0.53
COPD N (%)	30 (26.3)	36 (29.3)	0.35
DM N (%)	20 (17.5)	28 (22.8)	0.20
Cancer N (%)	45 (39.5)	67 (54.5)	< 0.001
Immunosuppression N (%)	14 (12.3)	8 (6.5)	0.09
ASA class III/IV N (%)	49 (43.0)	41 (33.3)	0.08
CeDAR Points (SD)	18.96 (14.0)	15.33 (9.9)	0.02

*AWS* abdominal wall surgeon, *GS* general surgeon, *COPD* chronic obstructive pulmonary disease, *BMI* body mass index, *DM* diabetes mellitus, *ASA class* American Society of Anesthesia Score, *CeDAR* Carolinas Equation for Determining Associated Risks score

Comparing hernia characteristics, we found no differences between groups in terms of location or grade of complexity. Moderate grade hernias were the most common in both groups, and there were more severe grade hernias in the AWS group without reaching statistical significance. Otherwise, hernias in the AWS group were larger (length > 10 cm; GS 5.6% vs. AWS 27.2%; *P* = 0.01;width > 10 cm; GS 13.8% vs. AWS 40.4%; P = 0.01), and more patients in the AWS group had a previous repair (GS 15.4% vs. AWS 26.3%; *P* = 0.02) (Table 2).

**Table 2:** Incisional Hernia Characteristics

	AWS *N* = 114	GS *N* = 123	*P* value
Location			
Midline N (%)	50 (43.9)	51 (41.5)	0.71
Trocar Umbilical N (%)	29 (25.4)	38 (30.9)	0.35
Parastomal N (%)	4 (4.6)	10 (8.1)	0.13
Subcostal N (%)	5 (4.4)	6 (4.9)	0.86
Lumbar N (%)	3 (2.6)	0 (0)	0.07
Pfannenstiel N (%)	7 (6.1)	6 (4.9)	0.67
Others N (%)	16 (14.2)	12 (9.8)	0.30
Previous repair N (%)	30 (26.3)	19 (15.4)	0.02
Complexity^a^			
Minor N (%)	22 (19.3)	34 (27)	0.14
Moderate N (%)	65 (57)	69 (56.1)	0.31
Severe N (%)	27 (23.7)	20 (16.3)	0.13
Length > 10 cm N (%)	31 (27.2)	7 (5.6)	< 0.001
Width > 10 cm N (%)	46 (40.4)	17 (13.8)	< 0.001

^a^ Slater NJ et al. Hernia 2014; 18:7–17 [19]

Surgeries were performed mainly by senior surgeons in both groups (GS 61.8% vs. AWS 78.9%; *P* = 0.09). Comparison of repair techniques detected some differences. The most common technique in the GS group was suprafascial onlay mesh. The preferred type of mesh in the GS group was reticular polypropylene; whereas in the AWS group, it was composite. The sandwich technique (2 meshes in different layers) and abdominal components separation were used more frequently in AWS patients. Staples were the most frequent type of fixation in the GS group (56.8%); whereas, suturing was the most frequent type of fixation in the AWS group (97.4%) Some meshes were fixated combining suture and staples into general surgery group (Table 3).

**Table 3:** Characteristics of repair technique

	AWS *N* = 114	GS *N* = 123	*P* value
Mesh position			
Onlay N (%)	45 (39.5)	75 (62)	< 0.001
Sublay N (%)	66 (57.9)	37 (30.6)	< 0.001
Intraperitoneal N (%)	3 (2.6)	9 (7.4)	< 0.001
Type of mesh			
Reticular N (%)	49 (43)	74 (61.2)	< 0.001
Laminar N (%)	1 (0.9)	2 (1.7)	0.52
Composite N (%)	63 (55.3)	46 (38)	< 0.001
Mesh fixation			
Suture N (%)	111 (97.4)	63 (52.1)	< 0.001
Staples N (%)	17 (14.9)	68 (56.2)	< 0.001
Sandwich technique N (%)^a^	34 (29.8)	7 (5.8)	< 0.001
Anterior Components separation (ACS) N(%)^b^	24 (21)	2 (1.6)	< 0.001

^a^ two mesh repair in different lays positioning

^b^ Anterior component separation as described by Ramirez with mesh reinforcement

The length of surgery was longer into the AWS group, whereas in-hospital and postoperative complications and length of hospital stay were similar in both groups. Two patients in the AWS group died due to intestinal ischemia and acute heart failure, respectively (Table 4).

**Table 4:** Postoperative outcomes

Short-term outcomes (30 postop days)	AWS *N* = 114	GS *N* = 123	*P* value
Length of stay days (SD)	4.9 (10.15)	4.4 (7.27)	0.50
Operative time min (SD)	103.8 (73.21)	83.3 (52.58)	0.01
Intraoperative complications N (%)	2 (1.8)	2 (1.6)	0.94
Postoperative short term complications N (%)	26 (22.8)	36 (29.3)	0.26
Exitus N (%)	2 (1.8)	0 (0)	0.14
Hematoma N (%)	2 (1.8)	6 (4.9)	0.18
Seroma N (%)	13 (11.4)	16 (13)	0.71
Wound infection N (%)	8 (7)	11 (8.9)	0.58
Skin necrosis N (%)	1 (0.9)	3 (2.4)	0.35
Urinary infection N (%)	2 (1.8)	4 (3.3)	0.46
Respiratory infection N (%)	2 (1.8)	4 (3.3)	0.46
Other complications N (%)	5 (4.4)	8 (6.5)	0.47
Reoperations N (%)	1 (0.9)	4 (3.3)	0.20
Follow-up outcomes (min. 1 year)	*N* = 92 (80.7)^a^	*N* = 83 (67.7)^a^	*P* value
Chronic infection N (%)	0	1 (0.6)	0.47
Mesh removal N (%)	0	0	
Recurrence N (%)	11 (12)	24 (28.9)	0.008

^a^ Percentage of completed follow-up from initial sample population

One-year follow-up was completed by 175 patients (median: 36.06 mo). The long- term complications showed no difference between groups. A higher percentage of recurrences was detected in the GS group compared with the AWS group (GS 28.9% vs. AWS 12.0%; *P* = 0.005) (Table 4). This difference was also significative excluding parastomal hernias (GS 26.5% vs. AWS 9.2%; *P* = 0.001).

**Fig. 1:**
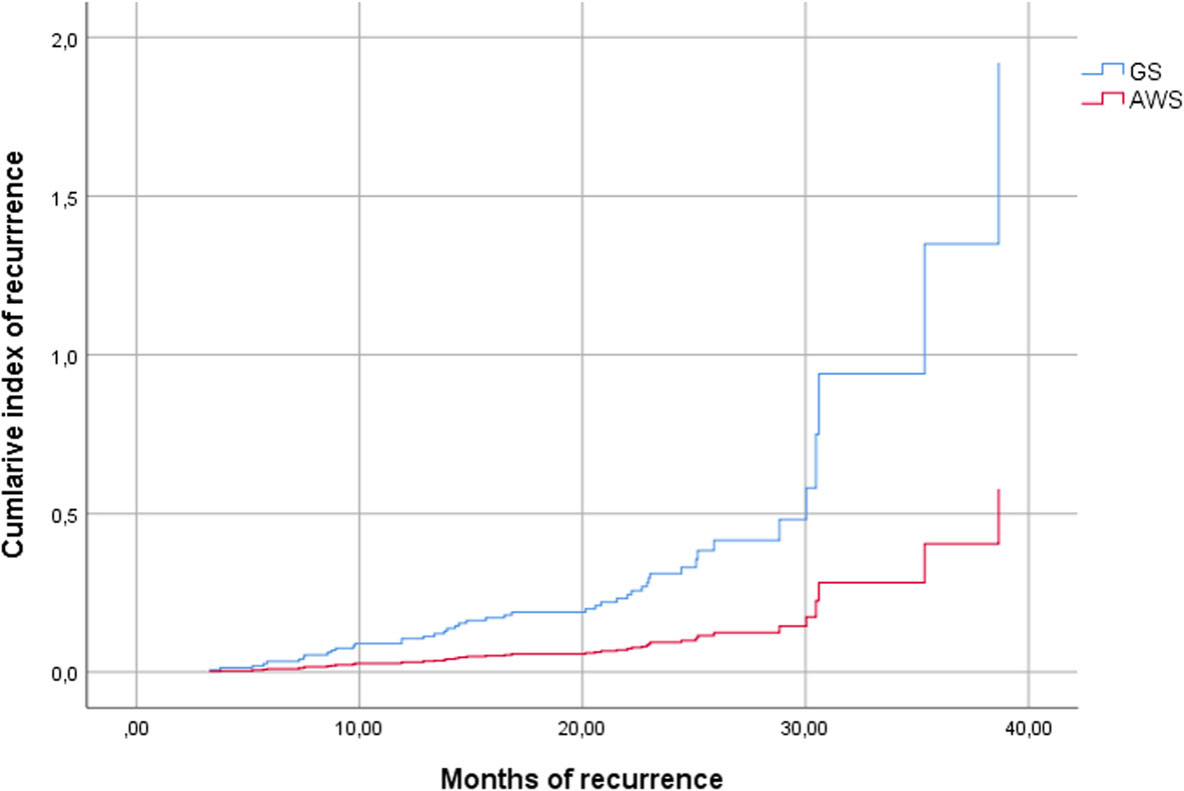
Cumulative incidence of recurrence

The cumulative incidence of recurrences using a Cox survival analysis was higher in the GS group (HR = 3.73; 95%; CI = 1.86–7.51) (Fig. 1).

Finally, using a multivariate analysis with a forward stepwise conditional logistic regression, only those surgeries performed by the AWS unit were related to less recurrence (OR = 0.19; 95%; CI = 0.07–0.58; *P* < 0.001; Otherwise, three factors were strongly related to higher recurrence: midline (OR = 5.81; 95%; CI = 2.16–15.63; *P* = 0.003) and parastomal location (OR = 2.47; 95%; CI = 2.16–15.63; P < 0.001) and previous hernia repair (OR = 3.21; 95%; CI = 1.26–8.14; *P* = 0.01) (Table 5).

**Table 5:** Multivariate analysis of risk factors for recurrence

	B	OR	Wald	95%CI	*P* value
AWS unit	−1.61	0.20	8.75	0.07–0.58	0.003
Midline location	1.76	5.81	12.14	2.16–15.63	< 0.001
Parastomal location	2.47	11.86	4.67	1.26–111.82	0.031
Sutured mesh	−0.93	0.39	3.20	0.14–1.09	0.073
Previous repair	1.35	3.85	6.95	1.14–10.50	0.008

## Discussion

Specialization in General and Digestive Surgery is common in some areas such as Colorectal Surgery, Hepatobiliary and Pancreatic Surgery, Gastrointestinal Surgery, Obesity Surgery, Emergency Surgery, Surgical Oncology, Breast Surgery, and Endocrine Surgery, and this specialization is regulated and audited by boards using examinations and practice requirements [22]. In previous papers [23, 24], better outcomes have been demonstrated in high-level centers and by specialized surgeons. However, although some suggestions have been made [15, 16], until now abdominal wall surgery has had no specific regulation for specialization or to certify expertise.

Our study shows that surgeon specialization in abdominal wall surgery is one main factor to reduce recurrences in open elective incisional hernia repair. This fact also has been described in the Shouldice technique for inguinal hernia repair, performed at the Shouldice Hospital, which has a four-fold decreased risk of recurrence compared with mesh repair performed in generalist hospitals in Canada [25]. Low recurrence rates also have been related to specific techniques for ventral hernia repair, such as preperitoneal ventral hernia repair [26], achieving a 5.2% rate of recurrence; and, reoperation rates for recurrence, operative time, and costs were lower in high-volume surgeons (> 36 operations/year) [27].

No differences between groups of patients were observed, except a high percentage of patients with previous oncological surgery in the GS group. These patients received surgery for a previous oncologic condition from the same surgeon who performed the hernia repair. For this reason, also, there are more parastomal hernia repairs in GS group, patients were operated by the same surgeon that performed previous stoma surgery.

The complexity and type of hernia were similar in both groups. However, a higher percentage of patients in the AWS group had a previous hernia repair and higher transversal and longitudinal diameters. Both factors point to a higher probability of recurrences and complications in this group [28], whereas in our study, the results showed fewer recurrences during follow-up.

The AWS group patients had a greater variability in the type of surgical techniques compared with the GS group. In our opinion, this fact could be related to a more tailored surgical approach and rational use of surgical resources in the AWS group. The GS surgeons more frequently used a reticular onlay mesh with staple fixation. These data are similar to those of our National Registry [5]. In our opinion, this suggests that more education in abdominal wall surgery should be provided in our country, and guidelines for the treatment of incisional hernias based on result of national and international registries should be developed. Further, membership on an international board for abdominal wall surgery should be mandatory for the leadership of specialized units.

The results of our study support the idea that elective incisional hernia repair should be performed by an Abdominal Wall Surgery Specialized Unit, and that the choice of the best surgical approach guided by expertise may be more important than the surgeon’s surgical performance.

The strengths of our study are that all data were collected prospectively and registered so they can be checked and audited. The weaknesses are that, by the time of the study, our center had available only one specialized surgeon, and there is no clear consensus of abdominal wall surgeon or unit definition. On the other hand, our unit and specialized surgeon had both accomplished the requirements for certified hernia centers suggested in Italy and Germany [15, 16].

## Conclusions

In conclusion, in this study, we found that any type of open elective incisional hernia repair performed by a specialized abdominal wall unit has a lower recurrence rate.

## Data Availability

The datasets generated during and/or analysed during the current study are available from the corresponding author on reasonable request.

## Abbreviations

ASA: American Society of Anesthesiologists
AWS: Abdominal wall surgeons
BMI: Body mass index
CeDAR: Carolinas Equation for Determining Associated Risks
COPD: Chronic obstructive pulmonary disease
DM: Diabetes mellitus
GS: General surgeons
IHR: Incisional hernia recurrence
